# Horses in Denmark Are a Reservoir of Diverse Clones of Methicillin-Resistant and -Susceptible *Staphylococcus aureus*

**DOI:** 10.3389/fmicb.2017.00543

**Published:** 2017-04-03

**Authors:** Md Zohorul Islam, Carmen Espinosa-Gongora, Peter Damborg, Raphael N. Sieber, Rikke Munk, Louise Husted, Arshnee Moodley, Robert Skov, Jesper Larsen, Luca Guardabassi

**Affiliations:** ^1^Department of Veterinary Disease Biology, Faculty of Health and Medical Sciences, University of CopenhagenFrederiksberg C, Denmark; ^2^Microbiology and Infection Control, Statens Serum InsititutCopenhagen, Denmark; ^3^Section for Bacteriology, Pathology and Parasitology, National Veterinary Institute, Technical University of DenmarkFrederiksberg C, Denmark; ^4^Højgård HestehospitalMorud, Denmark; ^5^Department of Biomedical Sciences, Ross University School of Veterinary MedicineBasseterre, West Indies

**Keywords:** horse, MRSA, MSSA, CC398, *mecA*, *mecC*, IEC, WGS

## Abstract

Denmark is a country with high prevalence of livestock-associated methicillin-resistant *Staphylococcus aureus* (MRSA) clonal complex (CC) 398 in pigs. Even though pig farming is regarded as the main source of human infection or colonization with MRSA CC398, 10–15% of the human cases appear not to be linked to pigs. Following the recent reports of MRSA CC398 in horses in other European countries and the lack of knowledge on *S. aureus* carriage in this animal species, we carried out a study to investigate whether horses constitute a reservoir of MRSA CC398 in Denmark, and to gain knowledge on the frequency and genetic diversity of *S. aureus* in horses, including both methicillin-resistant and -susceptible *S. aureus* (MSSA). Nasal swabs were collected from 401 horses originating from 74 farms, either at their farms or prior to admission to veterinary clinics. Following culture on selective media, species identification by MALDI-TOF MS and MRSA confirmation by standard PCR-based methods, *S. aureus* and MRSA were detected in 54 (13%) and 17 (4%) horses originating from 30 (40%) and 7 (9%) farms, respectively. Based on *spa* typing, MSSA differed genetically from MRSA isolates. The *spa* type prevalent among MSSA isolates was t127 (CC1), which was detected in 12 horses from 11 farms and represents the most common *S. aureus* clone isolated from human bacteremia cases in Denmark. Among the 17 MRSA carriers, 10 horses from three farms carried CC398 t011 harboring the immune evasion cluster (IEC), four horses from two farms carried IEC-negative CC398 t034, and three horses from two farms carried a *mecC*-positive MRSA lineage previously associated with wildlife and domestic ruminants (CC130 t528). Based on whole-genome phylogenetic analysis of the 14 MRSA CC398, t011 isolates belonged to the recently identified horse-adapted clone in Europe and were closely related to human t011 isolates from three Danish equine veterinarians, whereas t034 isolates belonged to pig-adapted clones. Our study confirms that horses carry an equine-specific clone of MRSA CC398 that can be transmitted to veterinary personnel, and reveals that these animals are exposed to MRSA and MSSA clones that are likely to originate from livestock and humans, respectively.

## Introduction

Over the last decade, livestock-associated methicillin-resistant *Staphylococcus aureus* (MRSA) belonging to clonal complex (CC) 398 has emerged worldwide in various animal species, especially in pigs as well as in people in contact with MRSA-positive animals (Petinaki and Spiliopoulou, 2012). Zoonotic transmission from pigs to humans has been substantiated by various epidemiological studies (Lewis et al., 2008; Köck et al., 2013). In Denmark the number of human infections caused by MRSA CC398 has increased steeply from 13 cases in 2009 to 240 cases in 2014, and decreased to 208 cases in 2015 (DANMAP, 2014, 2015). Presently, the CC398-associated *spa* types t034 and t011 account for 19% of all MRSA infections in this country (DANMAP, 2014, 2015), and the majority of cases are found in people with direct or indirect (household members) contact with pigs (Voss et al., 2005). However, a steady proportion of MRSA CC398 cases (10–15% of all cases and 37% of infections) occur in people that are not exposed to livestock (Lekkerkerk et al., 2012; DANMAP, 2014, 2015; Larsen et al., 2015), raising questions about the possible source of infection in these people.

Denmark has a population of ~5.7 million people and produces nearly 31 million pigs per year, according to data from 2015 (Statistics-Denmark, 2015a,b). Approximately 88% of the pig farms are positive for MRSA CC398^1^. New studies in Denmark in 2015 have found one poultry farm, five veal calf farms and eight mink farms MRSA CC398-positive (2, 5, and 8%, respectively) (DANMAP, 2015). Other potential animal reservoirs in Denmark have not been investigated. However, studies from other countries have shown MRSA CC398 carriage in horses (0.5–11%; Van den Eede et al., 2009, 2012) as well as a high prevalence (8–15%) of this CC amongst equine clinical MRSA isolates (van Duijkeren et al., 2010; Vincze et al., 2014). It appears that a recent shift has occurred in the epidemiology of MRSA in horses, with CC398 gradually replacing CC8 as the most common CC (Loeffler et al., 2009; Van den Eede et al., 2009, 2013; van Duijkeren et al., 2010). In addition to the noticeable spread of MRSA CC398 in horses, recent studies suggest that zoonotic transmission between horses and humans may be enhanced by the presence of the phage ΦSa3 in horse isolates (Cuny et al., 2015; Jung et al., 2017). This phage carries the human immune evasion gene cluster (IEC) that provides *S. aureus* protection from the immune system of humans and horses (Jung et al., 2017). Data on *S. aureus* carriage and infection in horses are largely biased in favor of MRSA and very little is not about the prevalence and genetic diversity of methicillin-susceptible (MSSA) strains.

The objectives of this study were (i) to investigate whether horses constitute a reservoir of MRSA CC398 in Denmark and (ii) to gain information on the frequency and genetic diversity of *S. aureus* in this animal species, including both MRSA and MSSA.

## Materials and methods

### Study population and sampling

Between April and August 2015, nasal swabs were collected from horses located at their farms of origin or immediately upon arrival at two veterinary clinics involved in the study, prior to entering the clinical environment. Horses were excluded from the study if they showed signs of an upper respiratory tract infection involving nasal discharge that could interfere with *S. aureus* isolation, if they had visited the horse clinic within 30 days of sampling or if the owner was not present to provide written consent to the sampling. The maximum number of horses included per farm was 20. Sampling was performed by veterinarians by inserting a sterile cotton swab moistened with Stuart's medium (BBL™ CultureSwab™, Becton Dickinson, U.S.A.) into the ventral meatus and the nasal vestibulum of both nostrils (Van den Eede et al., 2013). Samples were sent within 24 h to the Department of Veterinary Disease Biology, University of Copenhagen, and analyzed on the day of arrival.

A questionnaire filled by the horse owners was used to collect information about each horse including age, sex, current health status, and history of antimicrobial therapy in the last 6 months prior to sampling. Information was also retrieved about farm type (small private farm, riding school, or stud farm), presence of other animals on the farm (pigs, dogs, cats, cattle, sheep, or other) and the postal code. In addition to the questionnaire information, data on density of pigs was retrieved for each postal code where the horse farms were located. Number of pigs was retrieved from the Danish Central Husbandry Register (CHR) online database of the Danish Veterinary and Food Administration (Danish Veterinary and Food Administration)^2^ and postal code areas (sqKm) were provided by the Danish Geo Data Agency (Kortforsyningen)^3^.

The study was single-blinded meaning that the investigators were not informed about the origin of samples except for the postal code. This study did not require approval by an ethics committee according to the Danish Animal Experimentation Act §1.2.

### Isolation and characterization of *S. aureus*

Swabs were enriched 18–24 h at 37°C in 5 ml of Mueller Hinton broth (Sigma-Aldrich, U.S.A.) containing 6.5% NaCl. A 10 μl loopful of broth was plated both onto Brilliance™ MRSA2 agar (Oxoid, UK) and SaSelect™ agar (Bio-Rad, U.S.A) followed by incubation for 24 h at 37°C. One presumptive MRSA colony on Brilliance™ and up to three (if present) presumptive *S. aureus* colonies on SaSelect™ were sub-cultured onto blood agar. In order to increase the chance of detecting diversity, morphologically distinct colonies were chosen for sub-culturing from the SaSelect™ agar plates when possible. If no presumptive *S. aureus* was visible on SaSelect™ plates, these were then incubated for another 24 h. Isolates were tested by matrix-assisted laser desorption/ionization time-of-flight mass spectrophotometry (MALDI-TOF MS) (BioMérieux, France) for species confirmation. Confirmed *S. aureus* isolates were stored at −80°C for further analysis.

A multiplex PCR assay was performed to detect (i) *spa* encoding the staphylococcal protein A (Kahl et al., 2005), (ii) *mecA* and *mecC* encoding methicillin resistance (Oliveira and de Lencastre, 2002; Stegger et al., 2012), (iii) *scn*, a marker of IEC encoding staphylococcal complement inhibitor protein (*scn-F1*: 5′TACTTGCGGGAACTTTAGCAA3′ and *scn-R1*: 5′AATTCATTAGCTAACTTTTCGTTTTGA3′, amplicon size: 130 bp), (iv) the CC398-specific *sau1-hsdS1* variant (Stegger et al., 2011) using a newly designed forward primer *FP2sau1*: 5′GAGAATGATTTTGTTTATAACCCTAG3′ (amplicon size 106 bp), and (v) *lukF-PV*, a marker of the Panton-Valentine leucocidin (PVL) (Deurenberg et al., 2004). The PCR reactions were carried out in a final volume of 13 μl containing 1 × Qiagen Multiplex PCR Master Mix (Qiagen, Germany), 2 μM of each primer, and 1 μl of bacterial DNA. The following PCR conditions were used: initial denaturation at 94°C for 15 min, followed by 25 cycles consisting of denaturation (94°C for 30 s), annealing (59°C for 60 s), and extension (72°C for 60 s), and a final extension step at 72°C for 10 min. All *S. aureus* isolates were *spa*-typed as described previously (Harmsen et al., 2003). BURP cluster analysis of the *spa* types was performed in the Ridom StaphType software (Ridom GmbH, Germany) using default settings to deduce likely multi-locus sequence types of methicillin-susceptible *S. aureus* (MSSA) isolates. The staphylococcal cassette chromosome *mec* (SCC*mec*) types and subtypes were determined by PCR for all CC398 isolates (Larsen et al., 2016a). The IEC type was determined by the presence of the *scn, chp, sak, sea*, and *sep* genes (van Wamel et al., 2006).

### Whole genome sequencing (WGS) and phylogenetic analysis of MRSA CC398 isolates

DNA was extracted with the DNeasy Blood & Tissue kit (Qiagen, The Netherlands) and run on an Illumina MiSeq sequencer (Illumina, USA) for 250 bp paired-end sequencing. For comparative purposes, we included in the WGS-based phylogenetic analysis 16 porcine MRSA CC398 isolates from a previous Danish study (Grøntvedt et al., 2016) and 11 human MRSA CC398 isolates from the national MRSA database. The porcine isolates were selected to represent the three major phylogenetic groups in pigs in Denmark (Larsen, unpublished data). The human isolates included all IEC-positive t011 isolates obtained from humans in Denmark in the period from 2004 to 2015 (Table 1). Demographic data and information about risk factors for MRSA acquisition were obtained from written records and through telephone interviews. Use of these data has been approved by the Danish Data Protection Agency (protocol no. 2001-14-0021). Bioinformatic and phylogenetic analyses were performed as described previously (Larsen et al., 2016b). A previously published worldwide collection of 89 *S*. *aureus* CC398 isolates were included for comparative purposes (Price et al., 2012). The maximum-likelihood phylogeny was inferred from a total of 3,954 single-nucleotide polymorphisms (SNPs), of which 1,098 were parsimony-informative. We used 100 random bootstraps replicates for calculating branch support with PhyML 3.0 (Guindon et al., 2010). For illustration of the phylogenetic tree, we used R version 3.3.1 with packages ggtree (Yu et al., 2017) and ggplot2 (Wickham, 2009). Genomes were screened for specific genes by mapping sequence reads against reference sequences. References for *scn, chp, sak*, and *sea* were obtained from the sequence of *S. aureus* Newman (GenBank accession no. DQ530361); reference for *sep* was obtained from *S. aureus* COL (NC_002951); reference for vWbp Seq1 was obtained from *S. aureus* DL584 (HM228920); and reference for *lukP* and *lukQ* were obtained from *S. aureus* 3711 (LT671578). Genotypic resistance profiles were obtained with Mykrobe Predictor v0.4.3 build 0-gd6c8714 (Bradley et al., 2015).

**Table 1:** **Description of human individuals colonized or infected with IEC-positive *spa* type t011 MRSA CC398 in Denmark**.

**Case**	**Sex**	**Age, Y**	**Isolation year**	**Specimen**	**Epidemiologic findings**
A	F	38	2010	Screening (nares)	Wife of pig farmer (family A)
B	F	0	2010	Screening (nares)	Child of pig farmer (family A)
C	M	50	2011	Screening (nares)	Pig farmer
D	M	27	2014	Wound swab	Horse veterinarian (family B)
E	M	61	2014	Screening (nares)	Horse veterinarian (family B)
F	M	24	2014	Abscess	Pig farmer (family C)
G	F	32	2014	Wound swab	Horse contact
H	F	4	2014	Screening (perineum)	Niece of pig farmer
I	F	44	2014	Wound swab	Horse veterinarian
J	F	73	2015	Wound swab	No livestock contact
K	M	58	2015	Screening (nares)	Father of pig farmer (family C)

### Statistical analysis

**Figure 1:**
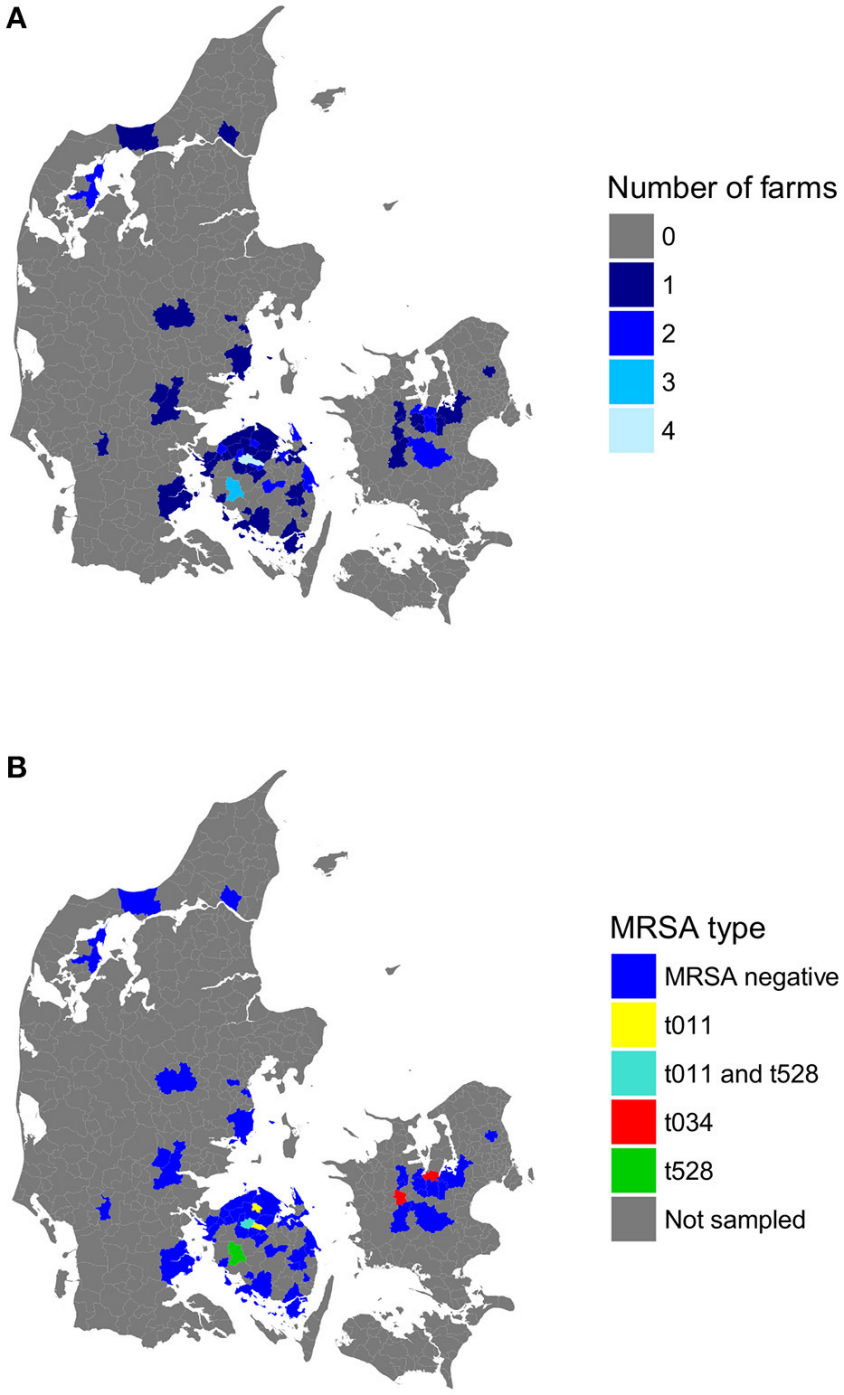
**Number of horse farms sampled (A)** and MRSA types detected **(B)** in each of the 53 postal codes included in the study. No farms were sampled for the postal codes colored in gray. Postal codes not associated with MRSA-positive farms are labeled as “MRSA-negative.”

Occurrence of total MRSA, MRSA CC398, and MRSA CC398 t034 (the only MRSA type of this study associated to pigs) were analyzed at the individual horse level and at the farm level by logistic analysis using the glmer function from the lme4 package (Bates et al., 2015) in R version 3.3.0 (RcoreTeam, 2016). For the horse-level analysis, age, sex, health status, farm type, and antimicrobial treatments were included as fixed effects; and farm was included as a random effect. For the farm-level analysis, farm type, presence of dogs, cats, cows, pigs, other species, postal code, density of pigs, and pig farms in the postal code were included as fixed effects. The 95% confidence interval of the prevalence values were calculated by the modified Wald method using the Graph Pad software Quick Calcs (GraphPad)^4^. Figure 1 was generated using the mapDK (Barfort, 2015) and ggplot2 (Wickham, 2009) packages in R 3.3.0 (RcoreTeam, 2016).

### Data access

The whole-genome sequence data from this study have been submitted to the NCBI Sequence Read Archive under BioProject PRJEB19362.

## Results

A total of 401 horses originating from 74 farms (41 small private farms, 20 riding schools, and 13 stud farms) were included in the study. The number of horses sampled per farm ranged from 1 to 20 with a median value of four. The sampled population consisted of 211 females and 190 males with a median age of 9 years (range: 3 months to 28 years). Three hundred and ninety-five horses were sampled on the farm where they lived, and six horses were sampled upon arrival to the clinic. Of them, 393 horses did not display any clinical signs at the time of sampling. The remaining eight horses had either laminitis (*n* = 2), colic/intestine alterations (*n* = 2), Cushing's syndrome (*n* = 1), dermatitis (*n* = 1), infection of the hoof (*n* = 1), or lung infection (*n* = 1). Three hundred and fifty-five horses had not been treated with antimicrobial agents in the preceding 6 months. Among the remaining horses, antimicrobial treatment was reported in 26 horses, and the treatment status was unknown for 20 horses (Data Sheet 1). The antimicrobial drugs used included trimethoprim/sulfadiazin (*n* = 12), cefquinome (*n* = 6), benzylpenicillinprocain (*n* = 3), ethacillin (*n* = 3), ampicillin (*n* = 1), doxycillin (*n* = 1), and tobramycin (*n* = 1). Three of the treated horses had received unknown antimicrobial drugs, and four had been treated with more than one drug. Most farms had cats (*n* = 52) and/or dogs (*n* = 51), three had cows, two had pigs, and five farms had other animals including alpaca, deer, and mouflon (*n* = 1), alpaca only (*n* = 1), hens (*n* = 1), rats/mice (*n* = 1), or fish (*n* = 1).

A total of 79 *S. aureus* (MRSA and MSSA) isolates were obtained from 54 horses (13.5%) originating from 30 farms (40.5%). MRSA was isolated from 17 horses (4.2%) from seven farms (9.5%) located in Funen and Zealand (Figure 1B; Table 2). The identified MRSA isolates belonged to three genotypes based on the PCR results: (i) IEC-positive MRSA CC398 t011 (10 horses from three farms), (ii) IEC-negative MRSA CC398 t034 (four horses from two farms), and (iii) IEC-negative *mecC-*MRSA CC130 t528 (three horses from two farms; Table 3). Each MRSA-positive farm harbored only one of these genotypes. One of the 17 MRSA carriers reported health problems (dermatitis) and unknown antimicrobial history, whereas the remaining 16 carriers were healthy and had received no antimicrobials in the previous 6 months.

**Table 2:** ***Staphylococcus aureus*, MRSA and MRSA CC398 prevalence in horses and horse farms**.

	**Horses (*n* = 401)**		**Horse farms (*n* = 74)**	
	**No. positive**	**Prevalence% (95% CI)**	**No. positive**	**Prevalence% (95% CI)**
*S. aureus*	54	13.5 (10.5–17.2)	30	40.5 (30.1–51.9)
MRSA	17	4.2 (2.6–6.7)	7	9.5 (4.4–18.5)
MRSA CC398	14	3.5 (2.1–5.8)	5	6.8 (2.6–15.2)

**Table 3:** **Characterization of MRSA and MSSA isolates from horses in Denmark**.

***spa* type**	***mecA*/*mecC***	**IEC**	**Associated ST/CC (ref)**	**No. positive horses**	**No. positive farms**	**Co-colonization with**
t011	*mecA*	+	ST398/CC398 (Nemeghaire et al., 2014)	10	3	MSSA t127^b^, t865, t15815
t034	*mecA*	−	ST398/CC398 (Crombé et al., 2012)	4	2	
	−	–	ST398/CC398 (Crombé et al., 2012)	2	1	MSSA t127
t127	−	−	ST1/CC1 (Battisti et al., 2010)	11	10	MRSA t528; MSSA t034, t549
	−	+	ST1/CC1 (Battisti et al., 2010)	1	1	MRSA t011
t528	*mecC*	−	ST130/CC130 (Espinosa-Gongora et al., 2015)	3	2	MSSA t127
t549	−	−	ST1660^c^ (Cuny et al., 2016)	5	3	MSSA t1508, t3043
t701	−	+	ST6/CC5 (Yan et al., 2012)	1	1	
t865	−	−	Unknown	1	1	MRSA t011
t1166	−	−	ST133/CC133 (Gharsa et al., 2015)	2	1	
t1294	−	−	Unknown	3	3	
t1508	−	−	Unknown	2	2	MSSA t549, t3043
t1943	−	+	ST5 or ST130/CC5 or CC130 (Donker et al., 2009)	1	1	
t2420	−	−	ST133/CC133 (Gómez et al., 2015)	1	1	
t3043	−	−	ST1660^c^ (Sieber et al., 2011)	3	2	MSSA t549, t1508
t4735	−	−	ST133/CC133^a^	1	1	
t5100	−	−	ST1/CC1 (Seidl et al., 2015)	1	1	
t15815	−	−	Unknown	2	1	MRSA t011
t15816	−	−	ST1660^a^^,^^c^	2	1	MSSA t1508

a *Based on clustering by BURP analysis*.

b *IEC-positive*.

c *Does not belong to a clonal complex*.

Based on PCR results, 50 of the 79 *S. aureus* isolates were MSSA belonging to 15 *spa* types and carried by 41 horses. The population structure of the MSSA isolates differed notably from that of MRSA isolates. None of the MSSA isolates belonged to CC398. The two most prevalent *spa* types were t127 (12 horses) and t549 (4 horses), which are associated to CC1 and ST1660, respectively (Table 3). Four *S. aureus* isolates were confirmed by MALDI-TOF but were lost and could not be typed. Two *spa* types (t15815 and t15816) were identified in this study for the first time. Most *S. aureus*-positive horses (48 of the 54) carried only one *spa* type, whereas six horses were found to simultaneously carry two unrelated *spa* types, either two MSSA or one MSSA and one MRSA (Table 4). In addition to the MRSA CC398 t011 isolates, three MSSA CCs harbored *scn*: CC1 t127 (*n* = 2), CC5 t701 (*n* = 1), and CC5/CC130 t1943 (*n* = 1). The BURP analysis grouped the *spa* types into five clusters and provided presumptive ST/CC information of two *spa* types without previously reported multi-locus sequence type: t4735 (clustering with t2420) presumably belonging to CC133 (Gómez et al., 2015), and t15816 (clustering with t549 and t3043) presumably belonging to ST1660 (Sieber et al., 2011; Cuny et al., 2016; Table 3). None of the isolates carried PVL.

**Table 4:** **Genotypic features of MRSA CC398 isolates included in this study**.

**Isolate ID**	**Species**	***spa* type**	**SCC*mec* type**	***scn***	***chp***	***sak***	***sea***	***sep***	**IEC type**	**SNP 309-2**	**Clade**
H2	Horse	t011	IV(2B)a	+	+	+	−	−	B	+	H
H42	Horse	t011	IV(2B)a	+	+	+	−	−	B	+	H
H43	Horse	t011	IV(2B)a	+	+	+	−	−	B	+	H
H44	Horse	t011	IV(2B)a	+	+	+	−	−	B	+	H
H46	Horse	t011	IV(2B)a	+	+	+	−	−	B	+	H
H155	Horse	t011	IV(2B)a	+	+	+	−	−	B	+	H
H157	Horse	t011	IV(2B)a	+	+	+	−	−	B	+	H
H160	Horse	t011	IV(2B)a	+	+	+	−	−	B	+	H
H161	Horse	t011	IV(2B)a	+	+	+	−	−	B	+	H
H163	Horse	t011	IV(2B)a	+	+	+	−	−	B	+	H
Case D	Human	t011	IV(2B)a	+	+	+	−	−	B	+	H
Case E	Human	t011	IV(2B)a	+	+	+	−	−	B	+	H
Case I	Human	t011	IV(2B)a	+	+	+	−	−	B	+	H
H223	Horse	t034	V(5C2&5)c	−	−	−	−	−	−	−	P1
H224	Horse	t034	V(5C2&5)c	−	−	−	−	−	−	−	P1
Case F	Human	t011	V(5C2&5)c	+	+	+	+	−	A	−	P1
Case H	Human	t011	V(5C2&5)c	+	+	+	+	−	A	−	P1
Case J	Human	t011	V(5C2&5)c	+	+	+	-	−	B	−	P1
Case K	Human	t011	V(5C2&5)c	+	+	+	+	−	A	−	P1
55-100-009	Pig	t034	V(5C2&5)c	−	−	−	−	−	−	−	P1
55-100-067	Pig	t011	V(5C2&5)c	−	−	−	−	−	−	−	P1
55-100-079	Pig	t034	V(5C2&5)c	−	−	−	−	−	−	−	P1
55-100-124	Pig	t034	V(5C2&5)c	−	−	−	−	−	−	−	P1
55-100-133	Pig	t034	V(5C2&5)c	−	−	−	−	−	−	−	P1
55-100-016	Pig	t011	V(5C2&5)c	−	−	−	−	−	−	−	P2
55-100-026	Pig	t011	V(5C2&5)c	−	−	−	−	−	−	−	P2
55-100-056	Pig	t011	V(5C2&5)c	−	−	−	−	−	−	−	P2
55-100-080	Pig	t034	V(5C2&5)c	−	−	−	−	−	−	−	P2
55-100-132	Pig	t011	V(5C2&5)c	−	−	−	−	−	−	−	P2
H280	Horse	t034	V(5C2)^*^	−	−	−	−	−	−	−	P3
H285	Horse	t034	V(5C2)^*^	−	−	−	−	−	−	−	P3
Case A	Human	t011	V(5C2&5)c	+	+	+	−	−	B	−	P3
Case B	Human	t011	V(5C2&5)c	+	+	+	−	−	B	−	P3
Case C	Human	t011	V(5C2&5)c	+	+	+	−	−	B	−	P3
Case G	Human	t011	V(5C2)b	+	+	+	−	−	B	−	P3
55-100-042	Pig	t034	V(5C2&5)c	−	−	−	−	−	−	−	P3
55-100-073	Pig	t034	V(5C2&5)c	−	−	−	−	−	−	−	P3
55-100-081	Pig	t011	V(5C2&5)c	−	−	−	−	−	−	−	P3
55-100-087	Pig	t034	V(5C2&5)c	−	−	−	−	−	−	−	P3
55-100-096	Pig	t034	V(5C2&5)c	−	−	−	−	−	−	−	P3
55-100-137	Pig	t034	V(5C2&5)c	−	−	−	−	−	−	−	P3

*All isolates were negative to vWbp Seq1, lukP, lukQ*.

* *SCCmec subtype non-typeable*.

**Figure 2:**
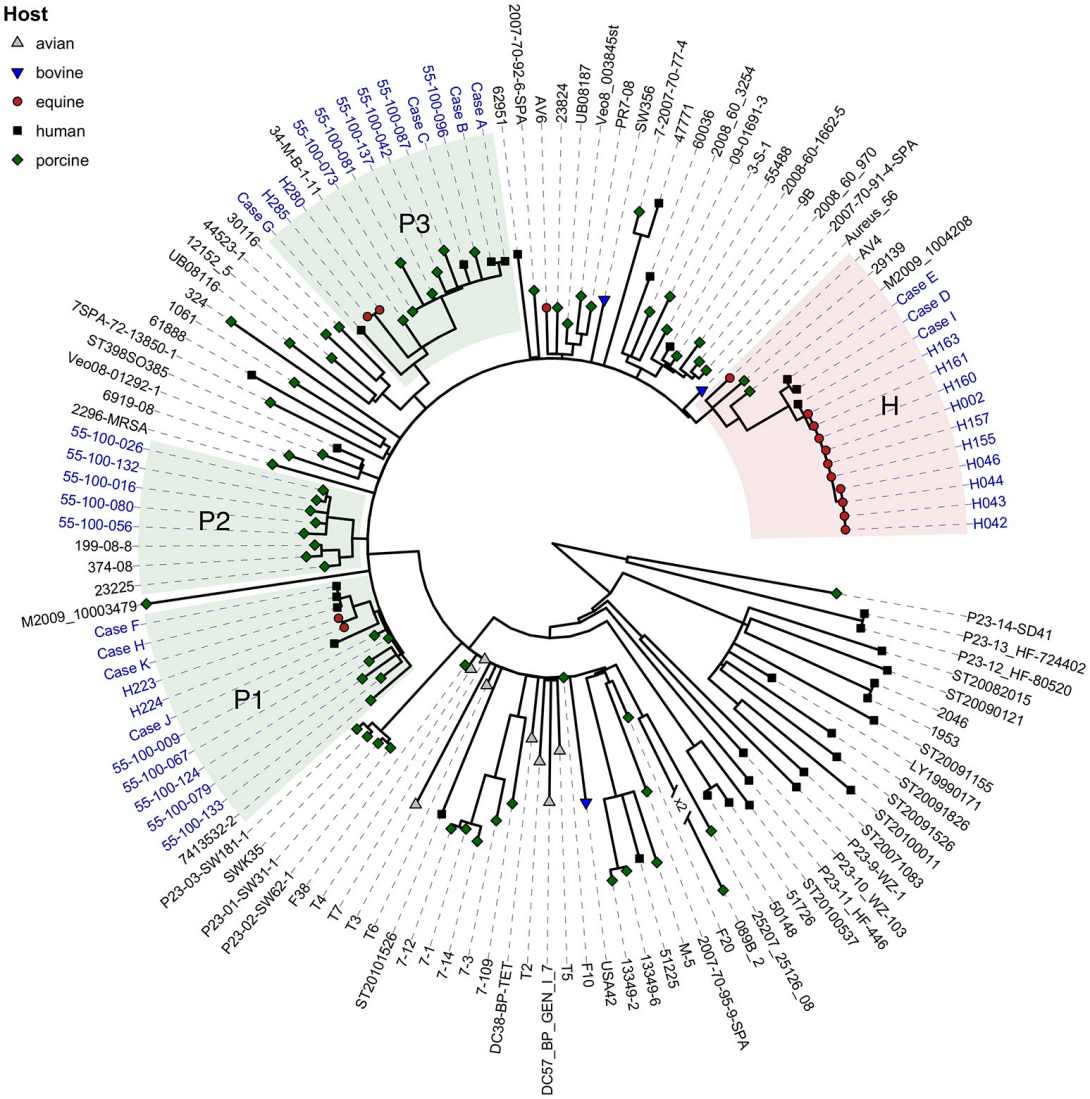
**Maximum likelihood phylogeny of MRSA CC398 isolates from Denmark (blue label) and from a worldwide collection (black label; Price et al., 2012)**. The maximum-likelihood phylogeny was inferred from a total of 3,954 single-nucleotide polymorphisms (SNPs), of which 1,098 were parsimony-informative. Color and shape of the branch endpoints indicate sample origin (see Legend). The three widespread Danish pig clones (clades P1–P3) are highlighted in green and the red-shaded area (clade H) highlights the isolates with the horse-specific marker SNP 309-2. The tree is midpoint rooted and numbers in broken branches indicate reduction factor in individual branch length.

Table 4 lists the genotypic characteristics of the MRSA CC398 isolated in this study. The four equine MRSA CC398 isolates with *spa* type t034 carried SCC*mec* V(5C2&5)c, lacked IEC, and clustered with two of the three widespread porcine clones in Denmark (hereafter referred to as pig-associated MRSA CC398). The 10 MRSA t011 from horses carried SCC*mec* type IV(2B)a and IEC type B, and formed a separate branch in the maximum-likelihood phylogeny (hereafter referred to as horse-adapted MRSA CC398; Figure 2; Table 4). Three of the 11 human MRSA t011 IEC-positive isolates form a monophyletic branch with 100% bootstrap support (Supplementary Image 1) together with all equine isolates with *spa* type t011. These isolates originated from equine veterinarians, including two members of the same family. The remaining eight human isolates either grouped together with, or were nearest neighbors to the IEC-negative *spa* type t034 isolates from pigs and horses (Figure 2; Table 4), and originated from two pig farm workers, four family members of farm workers, one person with contact to horses, and one person without any contact to livestock. The 10 horse isolates and the three closely related human isolates carried the unique SNP called 309-2 (Table 4). We also found SNP 309-2 in three IEC-negative MRSA CC398 isolates from the worldwide collection, including two porcine *spa* type t011 isolates from Hungary and Italy and one equine *spa* type t1451 isolate from Belgium. These three IEC-negative isolates were located on the same branch as, but basal to, the IEC-positive horse-adapted isolates from Denmark (Figure 2), suggesting that IEC was acquired after diversification of the horse-adapted clone.

All horse and human isolates within the horse-specific clade carried *aacA-aphD*, the gentamicin and kanamycin resistance determinant (Data Sheet 2). The gene was otherwise only found in two livestock-associated MRSA isolates from the worldwide collection, including a porcine *spa* type t011 isolate from Italy and a bovine *spa* type t567 isolate from Belgium, which is the closest neighbor to the horse-adapted clone. None of the isolates within the horse-specific clade carried the genes encoding LukPQ.

Occurrence of total MRSA, MRSA CC398, or pig-adapted MRSA CC398 t034 showed no significant associations at the individual-level analysis with age, health status, and antimicrobial use in the preceding 6 months, or at the farm-level analysis with farm type, presence of other animals on the farm or density of pigs or pig farms in the same geographical location based on postal code (Supplementary Image 2).

## Discussion

We detected MRSA in 4.2% of the horses in the study population, which mainly included healthy horses without recent admission to clinics or antimicrobial treatment. This prevalence is higher than those reported by previous studies in healthy horses from Canada, Belgium, Ireland, and the UK (0–1.7%; Burton et al., 2008; Abbott et al., 2010; Maddox et al., 2012; Van den Eede et al., 2012). A previous Danish study conducted in 2005 failed to detect MRSA amongst 100 horses, including both healthy and diseased horses (Bagcigil et al., 2007). Although, the study populations are not fully comparable, these data suggest an increase in the prevalence of MRSA in the horse population in Denmark over the last decade. The recent spread of MRSA in horses potentially poses a health risk to equine veterinary practitioners, horse owners, and other people in contact with positive animals. It also poses risks to animal health as evidenced by the increasing prevalence of MRSA infections in horses worldwide (Cuny and Witte, 2016).

Our study shows that horses in Denmark are a reservoir for a variety of MRSA genotypes, including both horse-adapted and pig-adapted MRSA CC398 as well as MRSA CC130 harboring *mecC*. IEC-positive MRSA CC398 t011 harboring SNP 309-2, which is a marker of a previously identified horse-adapted clone (Abdelbary et al., 2014), constituted 59% of the equine MRSA isolates. This clone was recently shown to be the most common MRSA type in clinical samples from horses in central Europe (Abdelbary et al., 2014). The IEC-positive MRSA CC398 t011 isolates shared other characteristics with horse isolates from Germany and the Netherlands in that they carried SCC*mec* type IV(2B)a and the gentamicin and kanamycin resistance determinant, *aacA-aphD* (Cuny et al., 2008; van Duijkeren et al., 2010). The latter genotypic feature may reflect the widespread usage of gentamicin in horses.

Whole-genome phylogenetic analysis showed that three of the human isolates (one from healthy carriage and two from infections) were highly similar to those isolated in this study from horses (Figure 2; Table 4). Interestingly, all these isolates originated from equine veterinarians, indicating an occupational risk for MRSA CC398 carriage and infection in veterinary staff. The horse-adapted clone accounted for 0.2% (2/834) of all MRSA CC398 infections during 2004–2015, according to the national MRSA database. In a previous study by Abdelbary et al. (2014), the horse-adapted clone was detected in Austrian veterinary personnel but not in other humans. Danish horse veterinarians have previously been associated to a higher risk of MRSA carriage compared to people not exposed to horses (Moodley et al., 2008). The current MRSA control policy in Denmark includes screening and decolonization of people at risk of MRSA carriage upon hospital admission, e.g., pig farmers. Whether or not horse veterinarians should be included as another risk group requires further epidemiological evidence through studies that assess the prevalence of MRSA carriage and infection in people in contact with horses, or investigate the exposure to horses in MRSA-positive patients.

The equine IEC-negative MRSA CC398 t034 isolates were closely related to two of the three clones that are widespread among pigs in Denmark (Figure 2), suggesting a recent transmission between these two animal hosts. The origin of pig-adapted MRSA CC398 in the horses is unknown, since pigs were not present in any of the positive horse farms. The lack of contact with pigs suggests that horses could be a reservoir for the pig-adapted MRSA CC398 t034 in addition to pigs. Moreover, the presence of SNP 309-2 in the two porcine t011 isolates from Hungary and Italy suggests a possible jump of MRSA CC398 from pigs to horses, where it subsequently acquired IEC through acquisition of the ΦSa3 phage.

The *mecC*-positive MRSA CC130 t528 was detected for the first time in horses in Denmark. *mecC*-positive MRSA CC130 and CC49 belonging to other *spa* types have been isolated from horses in France and Germany (Haenni et al., 2015; Cuny et al., 2016). MRSA CC130 is known to have a broad host spectrum, mainly ruminants among domestic animals (Paterson et al., 2012; Loncaric et al., 2013; Monecke et al., 2013; Gomez et al., 2014). In Denmark, *mecC*-positive MRSA CC130 t528 has been recognized as a cause of human infections (Petersen et al., 2013) and as a commensal in the nasal cavity of cattle (García-Álvarez et al., 2011), small ruminants (Eriksson et al., 2013), and wild animal species at the Copenhagen Zoo (Espinosa-Gongora et al., 2015). Further insight into the phylogenetic relationship between these isolates could shed light on the possible role of horses in the evolution and dissemination of this MRSA clone.

The overall *S. aureus* carriage rate in the study population was 13.5%, which is slightly higher than the MSSA prevalence reported in healthy horses in Canada (7.9%; Burton et al., 2008). The most common MSSA lineage, CC1 t127, was isolated from 12 horses at 11 farms. This is a ubiquitous *spa* type that has previously been isolated from horse MRSA infections (Cuny et al., 2008), and in Denmark it is the most common *spa* type isolated from human cases of *S. aureus* bacteremia (DANMAP, 2015). The apparent lack of host-specificity of t127 and thereby potential zoonotic relevance requires confirmation by high-resolution genotyping methods such as the WGS approach used in this study for CC398 isolates. The second most common *spa* type, t549 (ST1660), is considered a horse-adapted *S. aureus* clone (Cuny et al., 2016).

No significant associations were found between MRSA carriage and the variables covered by the questionnaire (age, health status, recent antimicrobial therapy, farm type, presence of other animals on the farm, and density of pigs or pig farms in the area of the farm). Our data suggest that antimicrobial use is not a major risk factor for MRSA carriage in horses. The factors contributing to the recent spread of MRSA CC398 in the horse population in Europe remain unknown. Further epidemiological studies are needed to assess whether MRSA transmission may be enhanced by movement of horses and horse owners in relation to sport and breeding activities.

To conclude, horses can be healthy carriers of distinct MSSA and MRSA clones that are implicated in human infections. In addition to horse-adapted MRSA CC398, which is a rare cause of human infection, horses in Denmark are exposed to distinct MRSA clones that are likely to originate from other animal species (CC130 t528 and pig-adapted CC398 t034) as well as to a MSSA clone that is prevalent in human cases of *S. aureus* bacteremia in Denmark (CC1 t127). Further research is needed to determine the stability of MRSA and MSSA carriage in horses, to identify risk factors and elucidate mechanisms of transmission between equine farms, and to quantify the risk of infection in people exposed to horses.

## Author contributions

Design of the work: LG, AM, PD, JL; Acquisition, analysis, or interpretation of data: MI, CE, RNS, RM, LH; Drafting the work or revising it critically for important intellectual content: MI, CE, PD, RNS, AM, RS, JL, LG; Final approval of the version to be published (all authors).

## Funding

The study was funded by the Danish Pig Levy Fund (Svineafgiftsfonden), and the Knowledge Centre for Agriculture/Danish Pig Research Centre (SEGES). SEGES Horses and the Danish Equestrian Federation funded whole-genome sequencing of MRSA isolates obtained from horses.

### Conflict of interest statement

The authors declare that the research was conducted in the absence of any commercial or financial relationships that could be construed as a potential conflict of interest.

## Footnotes

^1^Danish Veterinary and Food Administration Fødevarestyrelsen. Available online at: https://www.foedevarestyrelsen.dk/Nyheder/Aktuelt/Documents/MRSA%20ekspertgruppe%20-%20resultatene%20forekomst%20af%20husdyr-MRSA%20i%20svin%202016.pdf.

^2^Danish Veterinary and Food Administration Fødevarestyrelsen, Denmark. Available online at: www.foedevarestyrelsen.dk/.

^3^Kortforsyningen Danish Geo Data Agency. Available online at: http://kortforsyningen.dk/.

^4^GraphPad Graph Pad Quick Calcs. Available online at: http://www.graphpad.com/quickcalcs/confInterval1/.

## Supplementary material

Data Sheet 1 **Information about the health and antimicrobial treatments of 49 horses**. The remaining 352 horses included in the study were reported as both, healthy and without any antimicrobial treatments in the 6 months prior to the study, and were hence excluded from this table.

Data Sheet 2 **Summary table of resistance profiles obtained from Mykrobe Predictor including responsible genes/variants found**.

Supplementary Image 1 **Maximum likelihood phylogeny of relevant clades containing bootstrap values**. The phylogeny is drawed as cladogram.

Supplementary Image 2 **Density of pigs (pigs/sqKm) in each of the 53 postal codes included in the study and the corresponding MRSA type(s) found**. Postal codes without identified MRSA-positive horses are labeled as “MRSA negative.”
