# Supplementary figures and images for: Horses in Denmark Are a Reservoir of Diverse Clones of Methicillin-Resistant and -Susceptible Staphylococcus aureus
Source: Front Microbiol. 2017 Apr 3;8:543. doi: 10.3389/fmicb.2017.00543 (PMC5376617; doi:10.3389/fmicb.2017.00543)

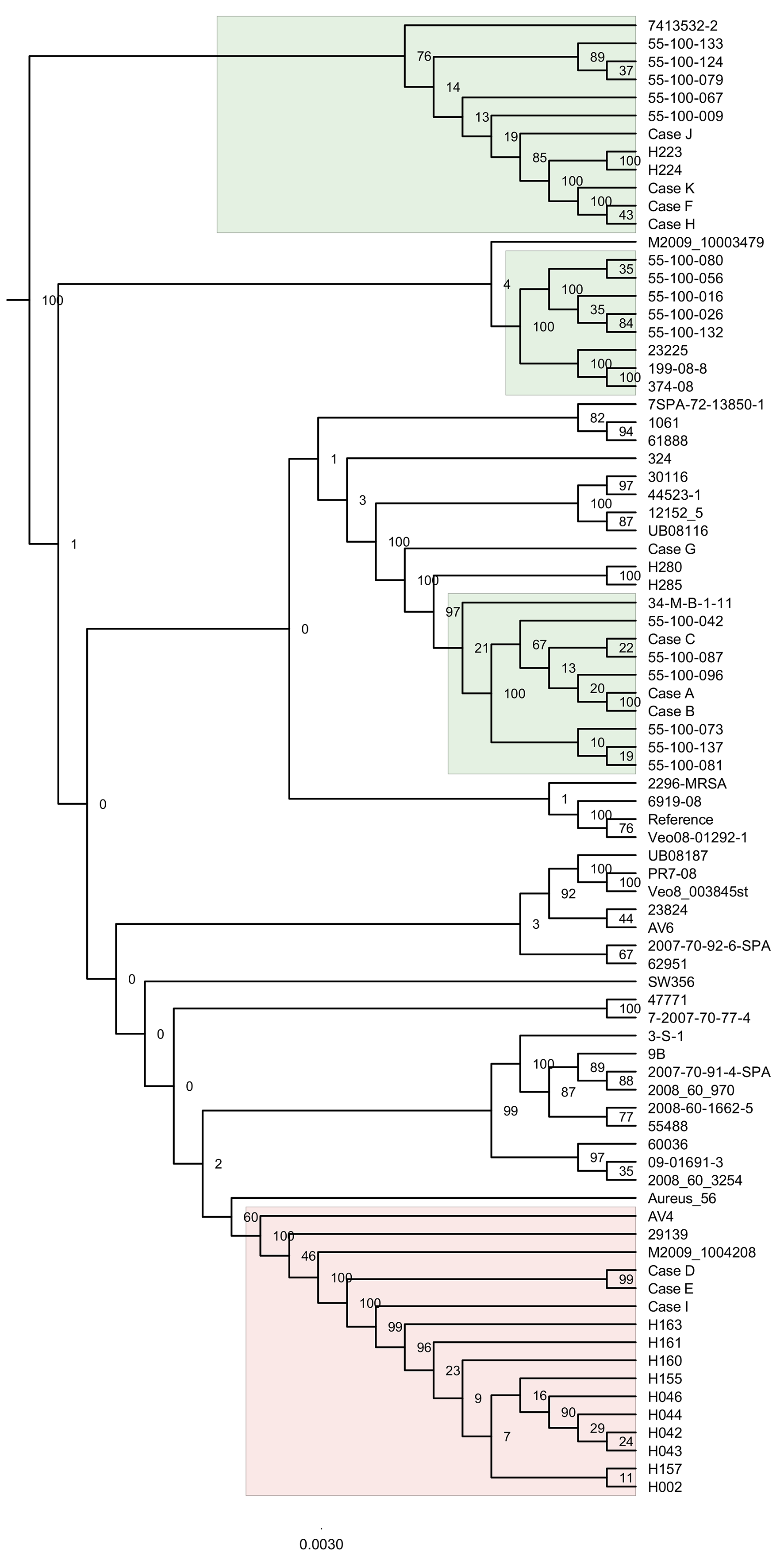

Supplement: Supplementary Image 1 — Maximum likelihood phylogeny of relevant clades containing bootstrap values. The phylogeny is drawed as cladogram. [file Image1.TIFF]

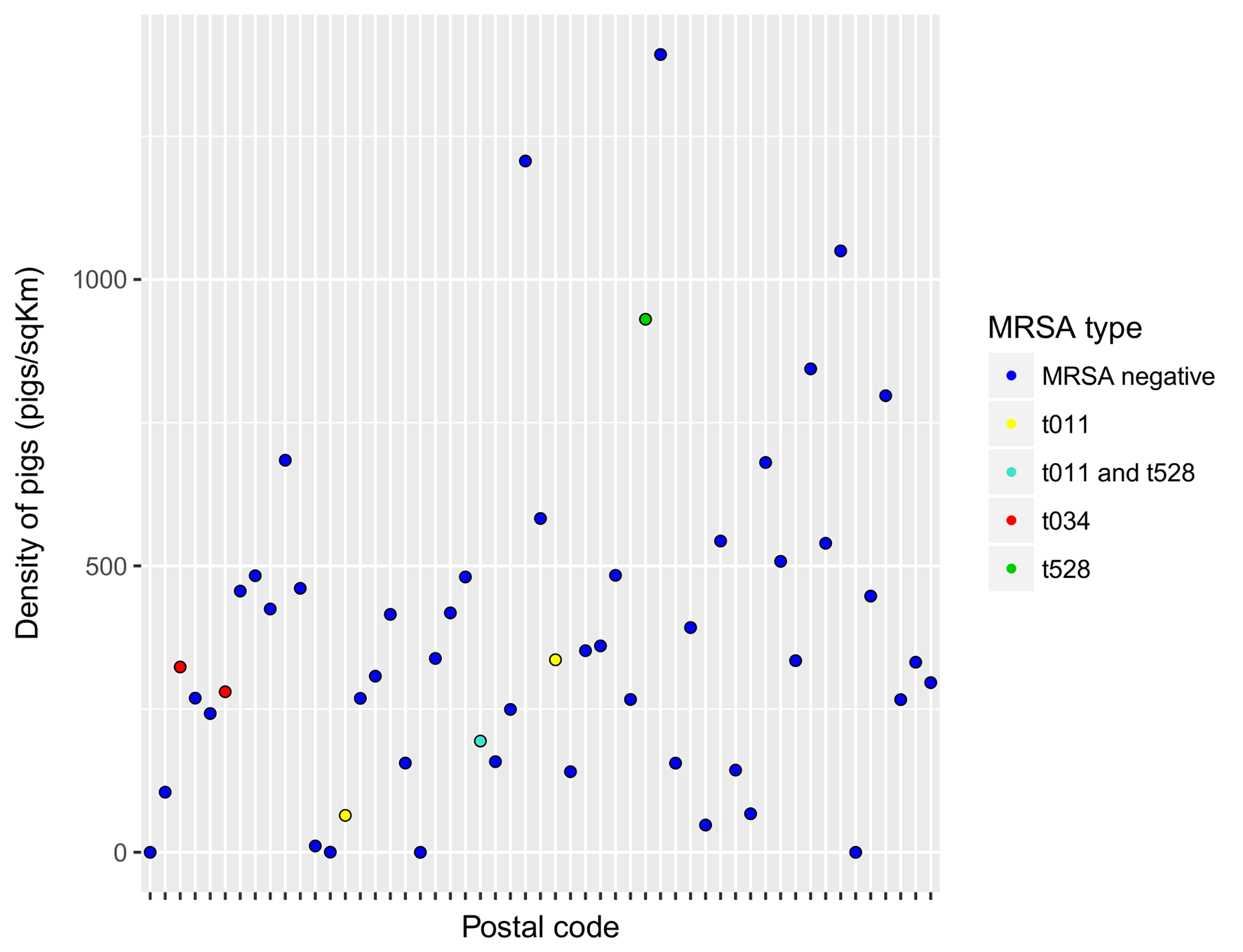

Supplement: Supplementary Image 2 — Density of pigs (pigs/sqKm) in each of the 53 postal codes included in the study and the corresponding MRSA type(s) found. Postal codes without identified MRSA-positive horses are labeled as “MRSA negative.” [file Image2.TIFF]
